# Olfactory Perception in Parkinson’s Disease: The Impact of *GBA1* Variants (Sidransky Syndrome)

**DOI:** 10.3390/ijms26115258

**Published:** 2025-05-30

**Authors:** Mikhal E. Cohen, Yosef Shechter, Melania Dominko, Elena Shulman, Tama Dinur, Shoshana Revel-Vilk, Roni Eichel, Gilad Yahalom, Michal Becker-Cohen

**Affiliations:** 1 Department of Neurology, The Eisenberg R&D Authority, Shaare Zedek Medical Center, Jerusalem 9103102, Israel; yosefs@szmc.org.il (Y.S.); melaniaka@szmc.org.il (M.D.); eichel@szmc.org.il (R.E.); gilady@szmc.org.il (G.Y.); 2 The Movement Disorders Unit, The Eisenberg R&D Authority, Shaare Zedek Medical Center, Jerusalem 9103102, Israel; 3 Faculty of Medicine, Hebrew University, Jerusalem 9112102, Israel; dinurt@szmc.org.il (T.D.); svilk@szmc.org.il (S.R.-V.); michalbc@szmc.org.il (M.B.-C.); 4 Gaucher Unit, The Eisenberg R&D Authority, Shaare Zedek Medical Center, Jerusalem 9103102, Israel; elename@szmc.org.il

**Keywords:** Parkinson’s disease, *GBA1* variant, Sidransky syndrome, smell, hyposmia

## Abstract

Parkinson’s disease (PD) associated with *GBA1* mutations—recently termed Sidransky syndrome—differs from idiopathic PD (iPD) by earlier onset, more rapid progression, and higher rates of non-motor symptoms. Our objective was to assess whether *GBA1* mutations contribute to olfactory dysfunction in PD and in asymptomatic carriers of the mutation. We compared olfactory and motor functions in 119 participants: Sidransky syndrome (n = 18), iPD (n = 30), *GBA1* variant carriers without PD (n = 21), Gaucher disease patients (n = 20), and healthy controls (n = 30). All were evaluated with the Brief Smell Identification Test (BSIT^®^) and the motor part of the Movement Disorders Society Unified PD Rating Scale (MDS-mUPDRS). Mean age was 59.2 ± 11.7 years. Mean disease duration was 2.5 ± 2.2 years in Sidransky syndrome and 5.4 ± 4.9 years in iPD. We found that both PD groups had significantly lower BSIT^®^ scores than non-PD groups (*p* < 0.001), particularly for leather, smoke, natural gas, pineapple, clove, rose, and lemon. Sidransky syndrome patients scored lower than iPD patients (*p* = 0.04). No significant olfactory deficits were observed in *GBA1* carriers or Gaucher patients without PD. We conclude that hyposmia is more pronounced in Sidransky syndrome than in iPD. However, normal olfaction in non-parkinsonian *GBA1* carriers suggests that *GBA1* variants alone do not account for olfactory loss in PD. Hyposmia likely reflects broader PD pathology rather than a direct effect of the *GBA1* mutation.

## 1. Introduction

The loss or reduction of the sense of smell, anosmia or hyposmia, is a well-known pre-motor symptom in Parkinson’s disease (PD), occurring sometimes years and even decades before the first appearance of motor symptoms of the disease [1,2,3], along with other symptoms such as sleep problems, depression, anxiety, or constipation [4].

The prevalence of hyposmia has been shown to differ between various genetic types of PD [5]. While the specific mechanisms causing hyposmia in PD are still debated, α-synuclein inclusion bodies have been detected along structures of the olfactory pathway [6].Moreover, the volume of the olfactory bulb and of other structures of the olfactory tract has been shown to be reduced in an imaging analysis of a PD patient’s brain [7,8]. Moreover, anosmia could predict cortical atrophy in PD [9].

Variants in the Glucocerebrosidase type 1 (*GBA1*) gene, which encodes the lysosomal enzyme glucocerebrosidase, are currently known as the most common genetic abnormality associated with PD [10]. Approximately 2–31% of PD patients carry a *GBA1* variant (*GBA1* carrier), in comparison to <1% of the healthy population [10]. The rate of *GBA1* associated PD *(GBA1*-PD) differs across populations, reaching 2–12% in non-Ashkenazi Jews versus 10–31% in Ashkenazi Jews [10].

The phenotype of *GBA1*-PD differs from the phenotype of idiopathic PD (iPD) by an earlier age of onset; on average, *GBA1*-PD is diagnosed 5 years earlier than iPD [11]. It causes a faster motor decline, particularly bradykinesia and axial impairment [12]. *GBA1*-PD patients present with a higher prevalence of non-motor symptoms including cognitive impairment and dementia, rapid eye movement (REM) sleep behavioral disorder (RBD) and autonomic failure [13,14,15]. Some reports suggest that brains from PD patients with *GBA1* mutations exhibit a more diffuse pattern of Lewy body distribution throughout the brain, compared to non-carriers [16], while other did not find any difference [17]. This typical entity of *GBA1*-PD has been recently suggested to be called Sidransky syndrome in honor of Ellen Sidransky’s significant contributions to the exploration of its clinical aspects. Sidransky syndrome is both autosomal recessive (when biallelic *GBA1* variants, i.e., Gaucher disease (GD)) and autosomal dominant (when monoallelic *GBA1* variants, i.e., *GBA1* carrier) and spreads from prodromal to full-blown PD.

Both patients with GD and *GBA1* carriers were shown to have impaired olfaction and some cognitive impairment compared to healthy controls [18]. However, in another study, no significant difference in olfaction was found between healthy controls, patients with GD, and *GBA1* carriers. The only statistically significant difference was found between patients with and without PD [19]. In the present study, we compared the smell perception in asymptomatic *GBA1* carriers, patients with Sidransky syndrome, and patients with iPD to healthy controls. We chose to concentrate on olfaction because it represents one of the earliest prodromal signs of PD, positioning it as a potential marker for predicting the onset and severity of the disease.

## 2. Results

### 2.1. Demography and Clinical Characteristics

**Figure 1:**
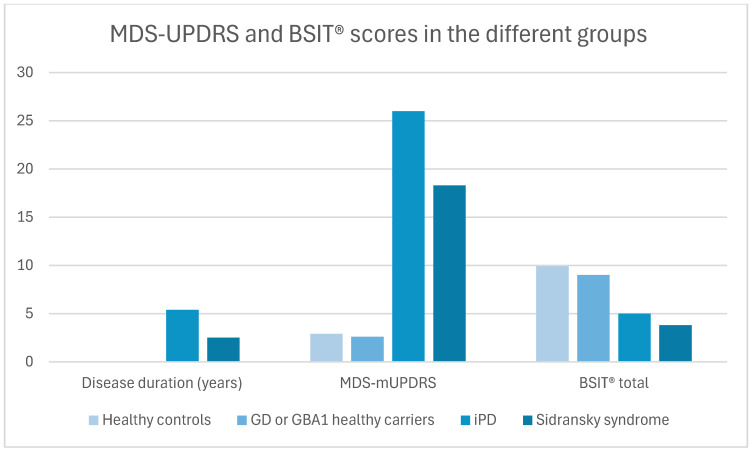
Disease duration, MDS-mUPDRS, and BSIT in the different groups. Abbreviations: MDS-mUPDRS= Movement Disorders Society motor part of the Unified Parkinson’s Disease Rating Scale; BSIT^®^ = Brief Smell Identification Test; GD = Gaucher disease; *GBA1* = glucocerebrosidase type 1; and iPD = idiopathic Parkinson’s disease.

A total of 119 participants (67 males) were recruited: 18 patients with Sidransky syndrome, 30 with iPD, 41 *GBA*1 carriers, and 30 healthy controls. The mean ± SD age was 59.2 ± 11.7 years, with patients with iPD being older than other groups (Table 1). The iPD group had a trend to a longer disease duration than the Sidransky syndrome group (*p* = 0.06). The MDS-mUPDRS score was higher in patients with PD (Sidransky syndrome and iPD) compared to the non-parkinsonian subjects (*p* < 0.001). The motor part of the Movement Disorders Society Unified PD Rating Scale (MDS-mUPDRS) was significantly higher in the iPD compared with Sidransky syndrome (*p* < 0.001). On a total of 12 fragrances of the Brief Smell Identification Test (BSIT^®^), the PD groups showed significantly lower scores than the non-parkinsonian groups (*p* < 0.001) (Table 1 and Figure 1). Due to the differences in age and disease duration between patients with Sidransky syndrome and iPD, a regression analysis adjusting for these variables was performed, showing that the total BSIT^®^ scores of patients with Sidransky syndrome were lower (*p* = 0.04) (Table 1).

### 2.2. Percentage of Identifications of the Different Smells Between the Groups

The two PD groups scored lower than the non-parkinsonian groups in all tested smells. However, following adjustment for age and sex, the PD groups (Sidransky syndrome and iPD) significantly under-detected the following smells: clove, leather, smoke, natural gas, pineapple, rose, and lemon, compared to *GBA*1 carriers and healthy controls (Table 2).

### 2.3. Difference in Smell Perception by Sex

Males showed lower scores than females in the smell perception of pineapple (*p* = 0.002), smoke (*p* < 0.001), and rose (*p* < 0.001) (Table 3).

### 2.4. Correlation Between Smell and Other Non-Motor Symptoms of PD

Total BSIT^®^ scores highly correlated with the self-report of impaired smell and inversely correlated with constipation (*p* = 0.001), RBD (*p* = 0.002), and MDS-mUPDRS (*p* < 0.001) (Table 4). Constipation correlated with urinary complaints (*p* = 0.005), RBD, and MDS-mUPDRS (*p* < 0.001 for each). MDS-mUPDRS correlated with urinary complaints (*p* < 0.001) and RBD (*p* = 0.034).

## 3. Discussion

This prospective study demonstrated that hyposmia was associated with a diagnosis of PD but not with being a non-parkinsonian *GBA1* carrier. In addition, in the comparison between patients with PD and non-parkinsonian participants, we might assume that the *GBA1* variant has no role in the cascade leading to smell impairment. The head-to-head comparison between Sidransky syndrome and iPD showed that after adjustments for age and disease duration, the *GBA1* variant may have a mild effect on smell perception, at least while PD is already diagnosed. The perception of smell became impaired as the disease progressed, and motor signs became more severe, as reflected by the correlation with MDS-mUPDRS. Also, the correlation of BSIT^®^ and other non-motor symptoms such as RBD and constipation suggests a decrease in smell perception as a predictor of disease severity.

From a mechanistic point of view, misfolded glucocerebrosidase produces stress on the protein degradation system. This causes endoplasmic reticulum stress which activates the unfolded protein response. The unfolded protein response prevents the alpha-synuclein from degrading at a normal pace leading to its aggregation and the formation of Lewy bodies [10]. Therefore, *GBA1* mutation induces olfactory dysfunctions through the induction of PD pathogenesis.

Research has shown that patients with *GBA1*-PD exhibit lower scores on the Hyposmia Rating Scale (HRS) compared to those with idiopathic Parkinson’s disease (iPD), with scores of 18.19 versus 19.55, respectively. Additionally, olfactory loss is more common in patients with *GBA1*-PD, affecting 54.48% of them compared to 40.95% of iPD patients. However, it is important to note that this study did not include asymptomatic carriers of *GBA1* variants (Liu et al., 2023) [12].

It was previously stated by Lopez et al. that the *GBA1* variant has no role in the cascade leading to smell impairment. They compared the smell perception of *GBA1* non-parkinsonian carriers to patients with Sidransky syndrome without including a control group and without comparing the smell scores to a group of iPD [19]. They also compared the total score of the University of Pennsylvania Smell Identification Test (UPSIT) but did not focus on differences in specific smells.

Following regression analysis, adjusted for age and sex, five smells were particularly difficult for patients with PD to identify: pineapple, natural gas, lemon, smoke, and leather. The lemon scent was especially challenging, as even healthy controls struggled to identify it, with only 50% success. Therefore, it can be omitted from the discussion. We attempted to identify a common factor in the misperception of smells within the PD population; however, explaining it proved challenging. The differences in smell perception may also be due to cultural habits. Indeed, previous studies on smell perception from different countries found varying results in smell discrimination [20,21,22].

This study had some limitations. The mean disease duration was 2.5 years for the Sidransky syndrome group and 5.4 years for the iPD group. In addition, the age of patients with iPD was significantly older than that of the other groups. These differences in age and disease duration can explain the higher MDS-mUPDRS score of the iPD group. Additionally, the sample size of the Sidransky syndrome group was relatively small. Since this study is retrospective, evaluations were performed in our clinic at different times of the day and without previous preparation.

## 4. Materials and Methods

**Figure 2:**
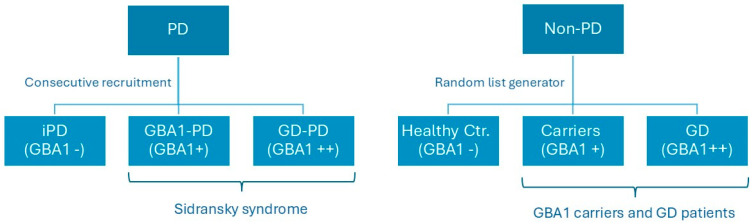
Recruitment of patients and controls of the study. Abbreviation: GD = Gaucher disease; *GBA1* = glucocerebrosidase type 1; PD = Parkinson’s disease; iPD = idiopathic Parkinson’s disease; and Ctr. = control.

Genetic testing was performed in all participants We screened patients for a panel of genes associated with PD (Appendix A)(CENTOGENE GmbH, Germany) *GBA1*-PD (Sidransky syndrome) patients and *GBA1* asymptomatic carriers tested positive for one *GBA1* mutation only. Other PD patients were negative for *GBA1* mutation. Gaucher patients with and without PD had biallelic mutations of *GBA1*. Healthy controls tested negative for the entire PD-gene panel (Figure 2). Consecutive patients with Sidransky syndrome and iPD followed at Shaare Zedek Medical Center Movement Disorder Clinic between 2020 and 2023 were included in the study. Data from a group of controls, who were not carriers for *GBA1* variants (n = 30), and *GBA1* carriers (n = 30) collected in a previous study [4] were incorporated into this analysis using a random list generator. Patients carrying other gene mutations associated with Parkinson’s disease were not included in the study. All groups were tested with version A of the Brief Smell Identification Test (BSIT^®^) (Sensonics international, Haddon Heights, NJ, USA) by two different testers (GY and MBC) and were evaluated by the motor part of the Movement Disorders Society Unified PD Rating Scale (MDS-mUPDRS) [11]. The examination included speech, facial expression, rigidity, finger tapping, hand movements, foot tapping, leg agility, posture, gait, postural stability, and tremor. Each item is rated on a scale from zero (normal) to four (severe impairment). The evaluation of the MDS-mUPDRS was performed by a movement disorders specialist (GY and MEC). Our trial was approved by the Institutional Review Board of Shaare Zedek Medical Center and was registered as trial no. 168-16 Version 3.0 on 19 September 2023. All participants signed an informed consent.

Anamnestic data on self-reports of impaired smell, constipation, and urinary complaints were collected. Rapid eye movements behavioral disorder (RBD) information was collected based on a single-question screen which showed a sensitivity of 93.8% and a specificity of 87.2%) [12].

### Statistical Analysis

Demographic data were analyzed using descriptive and frequency tables. Differences in continuous and dichotomous parameters between the groups were performed using, respectively, either one-way analysis of variance(ANOVA) or Chi-square tests. Head-to-head comparisons between Sidransky syndrome and iPD were calculated by a post-hoc least significant difference (LSD) test. For the total BSIT^®^ score, a comparison between Sidransky syndrome and iPD, was performed by a linear regression analysis, with adjustment for age and disease duration. As for the comparisons of smell identification among the subgroups, logistic regression was performed with adjustment for age and sex. The difference was defined as significant if the *p*-value was ≤0.05. In comparing the 12 smells, a correction for multiple variables was performed, and the *p*-value was defined as ≤ 0.004 (0.05/12). Pearson correlation was performed to compare correlations in variables. Analyses were performed using SPSS v. 29.

## 5. Conclusions

The *GBA1* variant seems to have no major role in the pathomechanism that leads to the development of hyposmia in PD. It is probably the disease itself that affects the olfaction. However, as PD is established, the *GBA1* variant might facilitate the deterioration of smell perception. Yet, it seems that the *GBA1* variant is associated with a worse course of PD, including smell perception, as part of other non-motor manifestations of PD, which deteriorate rapidly. This paper lays the foundation for a new eponym, Sidransky syndrome, which represents PD patients carrying a variant in one or both alleles in the *GBA1* gene.

## Figures and Tables

**Table 1:** Demographic and clinical characteristics of the different cohorts.

	GD or *GBA1* Healthy Carriers	Sidransky Syndrome	iPD	Healthy Controls	Total	*p*-Value
N	41	18	30	30	119	
Males	21	12	21	13	67	
Age, years (SD)	53.6 (9.8)	58.0 (11.0)	70.6 (8.2)	56.2 (9.7)	59.2 (11.7)	*p* < 0.001
Disease duration, years (SD)	NA	2.5 (2.2)	5.4 (4.9)	NA	4.6 (4.5)	0.12
MDS-mUPDRS	2.6 (3.8)	18.3 (8.5)	26.0 (9.5)	2.9 (3.3)	11.5 (12.3)	*p* < 0.001 * *p* = 0.02
BSIT^®^ total	9.0 (2.6)	3.8 (3.5)	5.0 (3.1)	9.9 (1.8)	7.4 (3.6)	*p* < 0.001 * *p* = 0.13 ** *p* = 0.02

The *p*-value for the comparison between all of the groups. * *p*-value for the comparison between Sidransky syndrome and iPD, following post-hoc one-way ANOVA test. ** *p*-value for the comparison between Sidransky syndrome and iPD, following linear regression. Abbreviations: N = number; MDS-mUPDRS= Movement Disorders Society motor part of the Unified Parkinson’s Disease Rating Scale; BSIT^®^ = Brief Smell Identification Test; GD = Gaucher disease; *GBA1* = glucocerebrosidase type 1; iPD = idiopathic Parkinson’s disease, and SD = standard deviation.

**Table 2:** A detailed smell performance in the BSIT among the different subgroups.

	GD or *GBA1* Healthy Carriers	Sidransky Syndrome	iPD	Healthy Controls	Total	*p*-Value	Adjusted for Age and Sex
Menthol	63.4	38.9	53.3	90.0	63.9	0.002	0.01
Cherry	78.0	27.8	46.7	80.0	63.0	<0.001	0.006
Clove	80.5	33.3	60.0	86.7	69.7	<0.001	0.003
Leather	87.8	44.4	40.0	93.3	70.6	<0.001	<0.001
Strawberry	58.5	33.3	26.7	76.7	51.3	<0.001	0.02
Lilac	73.2	27.8	43.3	76.7	59.7	<0.001	0.01
Pineapple	85.4	33.3	50.0	93.3	70.6	<0.001	<0.001
Smoke	95.1	38.9	46.7	90.0	73.1	<0.001	<0.001
Lemon	61.0	11.1	13.3	50.0	38.7	<0.001	<0.001
Soap	65.9	27.8	63.3	76.7	62.2	0.007	0.02
Natural gas	85.4	22.2	30.0	96.7	64.7	<0.001	<0.001
Rose	63.4	33.3	30.0	80.0	54.6	<0.001	0.004

Abbreviation: GD = Gaucher disease; *GBA1* = glucocerebrosidase type 1; and iPD = idiopathic Parkinson’s disease.

**Table 3:** A detailed smell performance in the BSIT by sex.

	**Males**	**Females**	**Total**	***p*-Value**
Menthol	53.7	76.9	63.9	0.007
Cherry	52.2	76.9	56.6	0.005
Clove	65.7	75.0	69.7	0.19
Leather	62.7	80.8	70.6	0.03
Strawberry	46.3	57.7	51.3	0.15
Lilac	55.2	65.4	59.7	0.18
Pineapple	56.7	88.5	70.6	<0.001
Smoke	61.2	88.5	73.1	<0.001
Lemon	34.3	44.2	38.7	0.18
Soap	55.2	71.2	62.2	0.06
Natural gas	55.2	76.9	64.7	0.01
Rose	38.8	75.0	54.6	<0.001

**Table 4:** Correlations between anamnestic hyposmia, quantitative anosmia, and other clinical variables.

		Total BSIT^®^	Constipation	Urinary COMPLAINTS	RBD	MDS-mUPDRS
Hyposmia	Pearson correlation	−0.672	0.172	0.063	0.100	0.185
	*p*-value	<0.001	0.354	0.733	0.620	0.320
	N	32	31	32	27	31
Total BSIT^®^	Pearson correlation	1	−0.354	−0.197	−0.313	−0.573
	*p*-value		<0.001	0.043	0.002	<0.001
	N	119	113	106	91	103
Constipation	Pearson correlation		1	0.273	0.447	0.483
	*p*-value			0.005	<0.001	<0.001
	N		113	103	89	98
Urinary complaints	Pearson correlation			1	0.312	0.371
	*p*-value				0.005	<0.001
	N			106	81	91
RBD	Pearson correlation				1	0.245
	*p*-value					0.034
	N				91	75

Abbreviations: BSIT = Brief Smell Identification Test; RBD = Rapid Eye Movement Behavioral Disorder; MDS-mUPDRS = Movement Disorders Society motor part of the Unified Parkinson’s Disease Rating Scale; and N = number.

## Data Availability

The data presented in this study are available on request from the corresponding author.

## Abbreviations

The following abbreviations are used in this manuscript:

ANOVA	Analysis of variance
BSIT^®^	Brief Smell Identification Test
GD	Gaucher disease
*GBA1*	Glucocerebrosidase type 1
*GBA1*-PD	*GBA1*-associated PD
iPD	Idiopathic Parkinson’s disease
IRB	Institutional review
LDS	Least significant difference
LSD	Least significant difference
MDS-mUPDRS	motor part of the Movement Disorder Society Unified Parkinson’s Disease Rating Scale
PD	Parkinson’s disease
*p*-value	Probability value
REM	Rapid eye movement (REM)
RBD	REM sleep behavioral disorder
SiS	Sidransky syndrome
SD	Standard deviation
SPSS	Statistical Package for the Social Sciences

## Appendix A

Panel depicting the list of genes analyzed.

**Gene**	**Name**
*ADCY5*	Adenylate cyclase 5
*ANO3*	Anoctamin 3
*APOE*	Apolipoprotein E
*APP*	Amyloid Beta Precursor Protein
*ATP13A2*	ATPase Cation Transporting 13A2
*ATP1A3*	ATPase Na+/K+ Transporting Subunit Alpha 3
*ATP9A*	ATPase Phospholipid Transporting 9A
*C19orf12*	Chromosome 19 Open Reading Frame 12)
*CHCHD2*	Coiled-Coil-Helix-Coiled-Coil-Helix Domain Containing 2
*CNEP1R1*	CTD Nuclear Envelope Phosphatase 1 Regulatory Subunit 1
*COX20*	Cytochrome c Oxidase Assembly Factor
*CTDNEP1*	CTD Nuclear Envelope Phosphatase 1
*DCTN1*	Dynactin Subunit 1
*DJ1 (PARK7)*	Parkinsonism-associated Deglycase
*DNAJC13*	DnaJ Heat Shock Protein Family (Hsp40) Member C13
*DNAJC6*	DnaJ Heat Shock Protein Family (Hsp40) Member C6
*ELOVL7*	ELOVL Fatty Acid Elongase 7
*FBXO47*	F-Box Protein 47
*FBXO7*	F-Box Protein 7
*GAK*	Cyclin G-Associated Kinase
*GBA*	Glucosylceramidase beta
*GCDH*	Glutaryl-CoA Dehydrogenase
*GCH1*	GTP Cyclohydrolase 1
*GNAL*	G Protein Subunit Alpha L
*GNE*	Glucosamine (UDP-N-Acetyl)-2-Epimerase/N-Acetylmannosamine Kinase
*GRN*	Granulin Precursor
*HPCA*	Hippocalcin
*KCTD17*	Potassium Channel Tetramerization Domain Containing 17
*KMT2B*	Lysine Methyltransferase 2B
*LPIN1*	Lipin 1
*LPIN2*	Lipin 2
*LPIN3*	Lipin 3
*LRRK2*	Leucine-rich Repeat Kinase 2
*MAPT*	Microtubule-associated Protein Tau
*MCCC1*	Methylcrotonoyl-CoA Carboxylase 1
*MCOLN1*	Mucolipin 1
*NPC1*	NPC Intracellular Cholesterol Transporter 1
*PANK2*	Pantothenate Kinase 2
*PARK2*	Parkinson Disease 2
*PDE8B*	Phosphodiesterase 8B
*PDGFB*	Platelet-derived Growth Factor Subunit B
*PDGFRB*	Platelet-derived Growth Factor Receptor Beta
*PINK1*	Mitochondrial Serine/Threonine-protein Kinase
*PLA2G6*	Phospholipase A2 Group VI
*POLG*	DNA Polymerase Subunit Gamma
*SLC19A3*	Solute Carrier Family 19 Member 3
*SLC20A2*	Solute Carrier Family 20 Member 2
*SLC30A10*	Solute Carrier Family 30 Member 10
*SLC39A14*	Solute Carrier Family 9 Member 14
*SLC6A3*	Solute Carrier Family 6 Member 3
*SNCA*	Synuclein Alpha
*SNCB*	Synuclein Beta
*SYN1*	Synapsin I
*SYNJ1*	Synaptojanin 1
*TAF1*	TATA-Box Binding Protein Associated Factor 1
*TDP43*	TAR DNA Binding Protein 43
*THAP1*	THAP Domain Containing 1
*TOR1A*	Torsin Family 1 Member A
*VAC14*	VAC14 Component Of PIKFYVE Complex
*VPS13C*	Vacuolar Protein Sorting 13 Homolog C
*VPS35*	Vacuolar Protein Sorting-associated Protein 35
*XPR1*	Xenotropic And Polytropic Retrovirus Receptor 1
